# Vaccinia virus A12L protein and its AG/A proteolysis play an important role in viral morphogenic transition

**DOI:** 10.1186/1743-422X-4-73

**Published:** 2007-07-11

**Authors:** Su Jung Yang, Dennis E Hruby

**Affiliations:** 1 Department of Microbiology, Oregon State University, Corvallis, Oregon 97331-3804, USA

## Abstract

Like the major vaccinia virus (VV) core protein precursors, p4b and p25K, the 25 kDa VV A12L late gene product (p17K) is proteolytically maturated at the conserved Ala-Gly-Ala motif. However, the association of the precursor and its cleavage product with the core of mature virion suggests that both of the A12L proteins may be required for virus assembly. Here, in order to test the requirement of the A12L protein and its proteolysis in viral replication, a conditional lethal mutant virus (*vvtetO*A12L) was constructed to regulate A12L expression by the presence or absence of an inducer, tetracycline. In the absence of tetracycline, replication of *vvtetO*A12L was inhibited by 80% and this inhibition could be overcome by transient expression of the wild-type copy of the A12L gene. In contrast, mutation of the AG/A site abrogated the ability of the transfected A12L gene to rescue, indicating that A12L proteolysis plays an important role in viral replication. Electron microscopy analysis of the A12L deficient virus demonstrated the aberrant virus particles, which were displayed by the AG/A site mutation. Thus, we concluded that the not only A12L protein but also its cleavage processing plays an essential role in virus morphogenic transition.

## Background

Proteolytic processing in vaccinia virus (VV) plays an important role in morphogenic transitions during the virus replication cycle. To date, six VV-encoded, proteolytically processed proteins have been reported. They are the gene products of A10L (p4a), A3L (p4b), L4R (p25K), A17L (p21K), G7L, and A12L (p17K) [1-6]. Extensive studies of these proteins have provided more specific mechanisms of VV proteolysis in terms of the transformation of immature virions (IV) into intracellular mature virions (IMV).

One of the VV major core proteins, A10L has been shown to be essential in virus replication and its absence in virus assembly resulted in defective virus morphology such as IV-like particles, which lacked granular viral materials and consequently produced the irregular-shaped virus particles [7]. These morphogenic defects suggested that A10L protein is required for the correct organization of the nucleocomplex within the IVs [7,8]. L4R, a DNA binding protein, plays an essential role in virus replication, being involved in an early stage of infection such as early transcription or unpackaging viral core and DNA [9,10]. The L4R-deficient virus produced virus particles with non-associated viroplasm and its surrounding viral membranes, suggesting its role in correct incorporation of viral DNA and cores with immature virus membrane.

On the other hand, both the G7L and A17L gene products, VV membrane proteins, are required for virus replication and are involved in the early development of IV membranes. G7L, a phosphoprotein in association with the A30L and H5R proteins, is responsible for the correct recruitment and attachment of crescent-shaped membranes to viroplasms [11]. The absence of G7L caused defective IV formation, which showed tubular elements apart from the granular virus materials as well as empty inside and multiple wrapped IV particles [5,12]. The A17L mutant virus under non-permissive conditions produced large aggregates of accumulated electron-dense materials and numerous vesicles/tubules engulfing viroplasms, demonstrating that A17L is an essential component for generation of IV and IMV membranes [13,14,5]. A17L (p21K) and its cleavage product (21K) co-localized with GTPase Rab1, a marker of intermediate compartment (IC) membranes, the origin of viral membrane [15] and demonstrated the A17L participation in very early stage of the membrane biogenesis. Thus, the researches on most of the VV structural precursor proteins that undergo proteolytic maturation elucidated that VV recruits and organizes the first recognized membrane and induces the correct formation of viral genome content through the proteolysis of viral core/membrane proteins. However, the essentiality and biological role of the A12L gene products still remained to be analyzed.

VV A12L is a late gene product, which is proteolytically processed from a 25kDa precursor (p17K) into a 17kDa cleavage product (17K) [4]. Its proteolysis is similar to the processing of the other VV core proteins in that the cleavage is sensitive to rifampicin, takes place at the conserved recognition motif, Ala-Gly-Ala (AG/A), and is associated with mature virions. On the other hand, unlike other core proteins, of which only the mature processed forms are localized to the virion, the fact that both p17K and 17K are observed in the core of mature virions suggests different regulation and participation of A12L proteolysis in virus assembly. In order to investigate the requirement of the A12L protein and elucidate its role in virion-morphogenesis, we constructed a conditional lethal mutant virus of A12L, of which protein expression can be regulated by tetracycline (Tet) [16]. The mutant virus was designed to have Tet operator in front of A12L open reading frame (ORF), where Tet repressors constitutively expressed from the T-REx 293 cell line bind to and block further transcription of A12L. The addition of Tet, however, prevents Tet repressors from binding to the Tet operator and switches on A12L expression. Here, we report that the absence of A12L results in approximately one log reduction of virus replication in concert with phenotypic defects. In addition, plasmid borne A12L with an N-terminal AG/A site mutation, which prevents A12L proteolysis, failed to rescue the A12L deficiency, demonstrating that A12L cleavage is essential for virus replication as well as formation of mature virions.

## Results

### Tet-regulated conditional mutant virus of A12L

**Figure 1:**
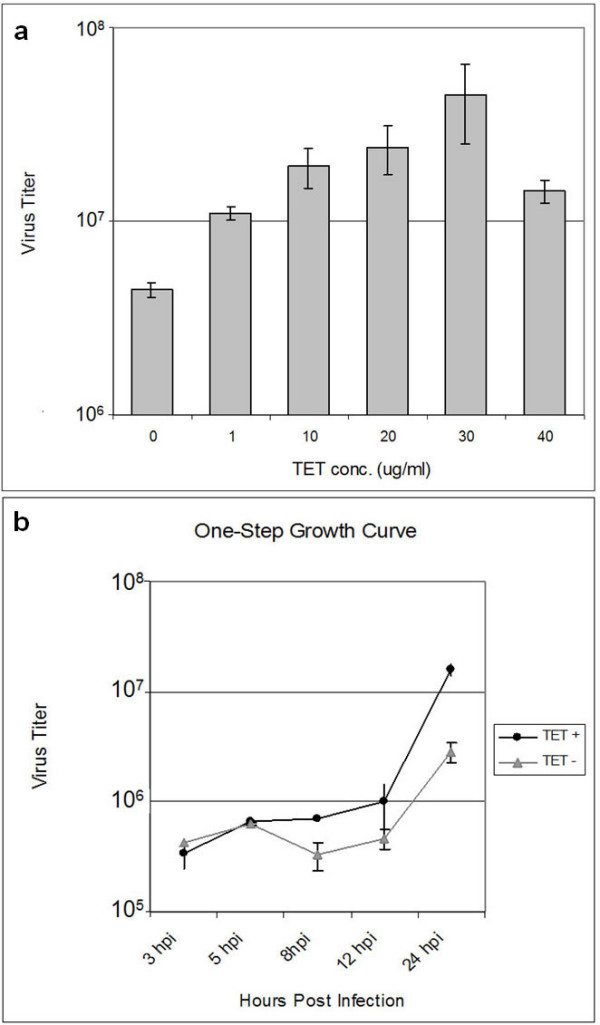
**Tet-dependent replication of *vvtetO*A12L and one-step growth curve**. a. Tet-dependent replication of *vvtetO*A12L. T-REx 293 cells were infected with *vvtetO*A12L at an MOI of 1 PFU/cell in the presence of tetracycline (Tet) at various concentrations of 0, 10, 20, 30, and 40 μg/mL. The infected cell extracts harvested at 24 hpi were titered on BSC 40 cells to determine the virus yields. b. One-step growth curve. T-REx 293 cells were infected with *vvtetO*A12L in the presence and absence of Tet (30 μg/mL) and harvested at 3, 5, 8, 12, and 24 hpi. Each virus titer (PFU/ml) was scaled in log phase.

To examine the regulation of a conditional mutant virus of A12L (*vvtetO*A12L), we infected T-REx 293 cells with *vvtetO*A12L at various concentrations of Tet from 0 to 40 μg/mL (Fig. 1a). Virus yield increased as the concentration of Tet increased from 0 to 30 μg/mL. This increased virus yield demonstrates that *vvtetO*A12L replicates in a Tet-dependent manner. Setting the optimal concentration of Tet at 30 μg/mL, we performed a one-step growth curve of *vvtetO*A12L with the cell extracts harvested at different time points after infection (Fig. 1b). The one-step growth curve shows the initial drop of virus yield at 5 hours post infection (hpi), when the A12L protein begins to be expressed as a late gene product. The maximum viral yields of *vvtetO*A12L in the presence of Tet was obtained at 24 hpi with approximately one log difference, which is attributed to the expression of the A12L protein and its essentiality in virus replication.

### Essentiality of A12L protein and AG/A cleavage in VV replication

**Figure 2:**
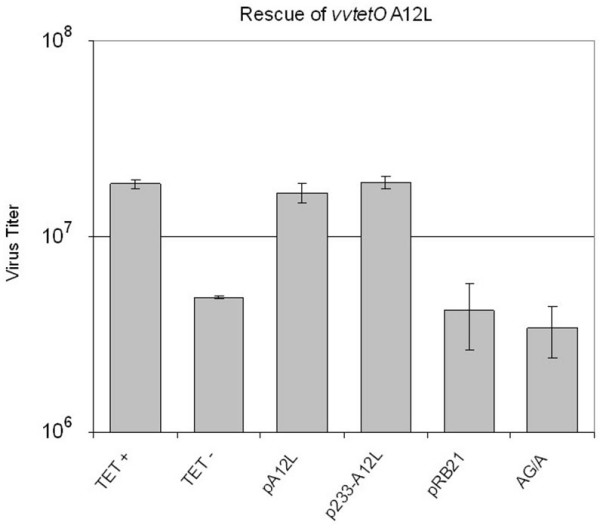
**Essentiality of A12L protein in VV replication**. In order to determine the essentiality of A12L protein in virus replication, T-REx 293 cells were infected with *vvtetO*A12L in the presence/absence of Tet (Tet+/-). The lack of A12L was complemented by the transient expression of plasmid born A12L under the control of an early/late synthetic promoter (pA12L) or the native promoter (233 nucleotide upstream of A12L ORF, p233-A12L). In addition, the N-terminal AG/A site mutated A12L was constructed to rescue the absence of A12L (AG/A). pA12L: A12L ORF under the control of the early/late synthetic promoter; p233-A12L: plasmid born A12L under the native promoter; pRB21: vector plasmid alone; AG/A: plasmid born A12L with N-terminal AG/A site mutation into ID/I. Each virus titer (PFU/ml) was scaled in log phase.

The sequence alignment of the A12L open reading frame with other representative orthopoxviruses such as cowpox, variola, and ectromelia viruses has shown highly conserved sequence alignment with more than 95 % identity (data not shown). Thus, it is expected that A12L may be essential for virus replication. An A12L conditional mutant virus (*vvtetO*A12L) was used to address the requirement of the A12L protein and the AG/A site cleavage for viral replication. To begin with, A12L protein expression was confirmed by immunoblot analysis with A12L specific bands obtained only in the presence of Tet (data not shown). Approximately 80 % reduction of virus titer was observed in the absence of Tet (Fig 2), suggesting that A12L plays an important role in viral replication. Confirmation that the defect in replication was due to the shut-off of A12L expression was obtained by a marker rescue experiment. Plasmid-borne A12L under the control of either its native promoter, which includes 233 nucleotides upstream of the A12L ORF (p233-A12L), or an early/late synthetic promoter in pRB21 vector (pA12L) provided almost 100% rescue in virus yield. This rescue experiment established the requirement of A12L expression in viral replication despite the leakiness of *vvtetO*A12L observed with the 80% viral reduction. Another rescue experiment of A12L expression with the AG/A site mutation (AG/A) into ID/I, however, failed to complement the absence of A12L protein, resulting in the similar virus yield to the titer of *vvtetO*A12L infection in the absence of Tet. Therefore, it is suggested that cleavage at the AG/A site plays an essential role in A12L functionality.

### Morphology defects in the absence of A12L expression

**Figure 3:**
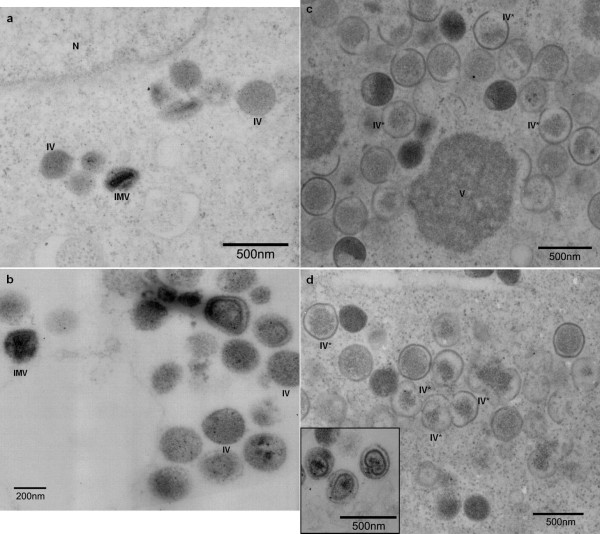
**Morphology defects in the absence of A12L expression**. To investigate a role of A12L protein in virus assembly, T-REx 293 cells were infected by *vvtetO*A12L in the presence (a, b) and the absence of Tet (c, d). In the presence of Tet, spherical IV particles were demonstrated, which evolved into the biconcave IMV particles. The inner layer of the core is localized along with the outer membrane (panel b). In the absence of Tet (c and d), mostly IV-like particles (IV*) were observed with accumulated viroplasms (V). IV-like particles contained little of viral dense materials in the membranes, which formed irregular-shape. Some of IV particles were developed into IMV-like particles, of which cores showed abrogated condensation along with abnormal-shaped layer as demonstrated in box at the panel d.

In order to study the phenotypic effects of A12L repression in virus assembly, T-REx 293 cells were infected with *vvtetO*A12L in the presence and absence of Tet (Fig. 3). In the presence of Tet, *vvtetO*A12L was able to assemble into mature virions as wild type VV does, producing oval particles with condensed cores (Fig. 3a–b). In the absence of Tet, *vvtetO*A12L displayed several phenotypic defects (Fig. 3c–d). The A12L deficiency caused accumulated granules of electron-dense areas including viral DNA and protein-rich aggregates (Fig. 3c) while crescent membranes were formed. Some immature virus particles (IV) were devoid of the internal materials or contained small IV contents surrounded by irregular-shaped membranes (IV-like particles, IV*). This indicates that the absence of A12L might delay or interrupt the viral membrane to adhere to the viral materials, which eventually led to the abrogated formation of spherical membranes. A small portion of the abnormal IV particles was able to mature into IMV but the core failed to form the characteristic of the bi-concave shape. Rather, the cores of the IMV retained a round shape, which appeared to lose the center-compressed concave structure. Thus, we concluded that the A12L deficiency led to not only the defects in the association of the viral contents with crescent-shaped membranes but also the formation of spherical IV membranes and subsequent disruption of interior cores of the IMV.

### Morphology defects by abrogated AG/A cleavage of A12L

**Figure 4:**
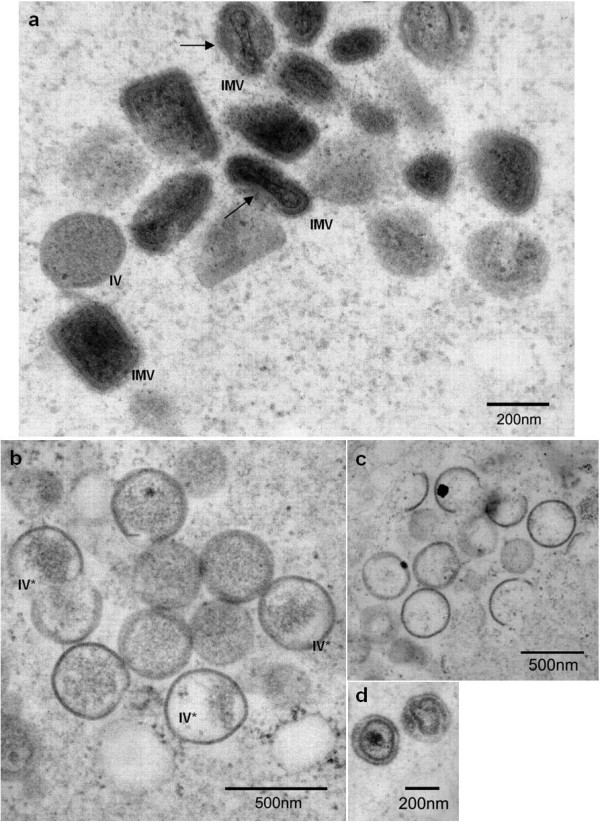
**Morphology defects by abrogated AG/A cleavage of A12L**. In order to examine VV morphology by rescuing the absence of A12L, we transfected plasmid born A12L under the control of an early/late synthetic promoter (pA12L) and AG/A mutant plasmid of A12L (AG/A), and infected with *vvtetO*A12L in the absence of Tet. The transient expression of A12L induced regular IV and IMV particles (panel a) while the AG/A mutation into ID/I displayed defective phenotypes (panel b through d). Arrows in panel a indicate center-concaved inner layer of the core. Panel b and c show IV particles with little or almost empty viral materials while panel d demonstrates the aberrant layers of the cores.

The morphogenic defects of the mutant virus under the restrictive conditions could be overcome by the transient expression of plasmid borne A12L (Fig 4a). Consistent with the rescue experiment, plasmid borne A12L (pA12L) was able to form regular IV particles, which had electron-dense viral materials inside and associated with the spherical membrane tightly. In addition, a condensed core was observed together with the development of the inner layer, which established the biconcave characteristics of IMV particles. The AG/A site mutated A12L, however, failed to produce fully matured IMV particles (Fig. 4b–d). Instead, the transient expression of AG/A site mutant A12L demonstrated similar phenotypic deformities as the absence of A12L, producing the irregular shaped IV-like particles with little viral material. Similarly, IMV particles retained round boundary membranes and abnormal inner layers (Fig. 4d). This can be explained by the fact that the impaired cleavage at an N-terminal AG/A site might lead to the improper core condensation and a concave inner core layer.

## Discussion

Here, we were able to report that the A12L deficiency is enough to delay viral replication as well as arrest the viral morphogenic transitions. Marker rescue experiments with pA12L and AG/A site mutated A12L (AG/A) not only confirmed the requirement of A12L in virus replication but also demonstrated that the disrupted A12L proteolysis eliminated its complementing functionality. This is also supported by the electron microscope analysis, which demonstrated the impaired morphological development of IV toward IMV by the failure of AG/A cleavage event.

The phenotypic defects such as detached viral membrane from the electron-dense virus materials, aberrant shape of IV particles, and disrupted bi-concave core layer of IMV particles suggest that A12L protein and its cleavage events may participate in the viral morphogenesis throughout from the early stage of IV formation to the very last stage of fully matured IMV. The abnormal IV-like particles similarly observed by the A10L deficiency imply that A12L may have a role in correct formation of nucleoprotein complex within the IV [7]. In addition, the abrogated biconcave IMV particles extend its role in the formation of a center-compressed core in IMV particles. In terms of the generation of viral membranes, A12L deficient virus introduced neither the absence of viral membrane nor unfinished or interrupted IV membranes, which were observed by the lack of A17L and A14L, respectively [17,18]. Thus, A12L protein is speculated not to be responsible for the generation of the crescent membranes but for their correct positioning and linkage to viroplasm. The similar phenotypic arrests obtained by the blocked AG/A site cleavage to the A12L deficient mutant virus may highlight the participation of VV proteolysis in the correct assembly of nucleoprotein complex in IV particles, the capability to maintain the stable spherical shape of IV, proper condensation of the core and its layer into center-concaved IMV formation. Therefore, additional characterization of the *vvtetO*A12L mutant virus will lead to the more specific biological function of the A12L protein during VV morphogenic transitions and regulation of A12L proteolysis.

## Conclusion

By demonstrating that A12L protein and its cleavage at an N-terminal AG/A play an important role in viral replication, we were able to conclude that all the VV core precursor proteins, which are proteolytically maturated, are required for the production of infectious progeny. The similar morphological defects observed by the A12L deficiency and single site mutation (AG/A) of A12L give emphasis to the significant participation of VV proteolysis in the viral morphogenic transition.

## Methods

### Cell cultures

Monolayer of BSC-40 cells was maintained in Eagle's minimal essential medium (EMEM, Invitrogen) supplemented with 10% fetal calf serum (FCS, Invitrogen), 2 mM glutamine (Invitrogen), and 10 mM gentamicin sulfate (Invitrogen) at 37°C in a 95% humidified atmosphere containing 5% CO_2_. For infection of the conditional mutant virus of A12L (*vvtetO*A12L), T-REx 293 cells (Invitrogen) were grown in Dulbecco's modified Eagle's medium (D-MEM, Invitrogen) supplemented with 10% Tet system approved fetal bovine serum (BD Biosciences), 2 mM Glutamax (Invitrogen), and 1% penicillin-streptomycin (Invitrogen), and incubated as described above. Blasticidin (5 μg/ml, Invitrogen) was added to the D-MEM growth media for selection of the pcDNA6/TR plasmid [19], which expresses the tetracycline repressors.

### Construction of conditional mutant virus of A12L (*vvtetO*A12L)

VV WR was used for the construction of the conditional mutant A12L virus (*vvtetO*A12L). The tetracycline operator (TetO) was inserted in front of the A12L ORF by virtue of two-step polymerase chain reaction (PCR) and amplified with 215 nucleotides (nts) upstream of the A12L ORF and 213 nts downstream of the A13L ORF. The PCR products were cloned into the p7.5:NEO vector [20], resulting in the construction of the p7.5:*TetOA12L*:NEO plasmid. Transfection of the p7.5:*TetOA12L*:NEO plasmid in concert with VV WR infection induced the first recombination. The Neomycin resistance gene (NEO^*R*^) in the p7.5:*TetOA12L*:NEO plasmid was used as a transient selective marker in the presence of Geneticin G418 sulfate (Invitrogen). The second recombination of NEO^*R*^-containing viruses occurred in the absence of Geneticin G418 sulfate, producing a wild type virus and an A12L mutant virus (*vvtetO*A12L) containing TetO without NEO^*R*^. Plaque purifications were performed in concert with PCR screens using the primers specific for TetO and 3' end of A12L ORF to identify pure *vvtetO*A12L isolates. Experimental infections of *vvtetO*A12L were carried out in T-REx 293 cell line to control the gene expression, which constitutively provides the Tetracycline repressor.

### Virus infections and titers

When T-REx 293 cells were approximately 80% confluent, *vvtetO*A12L virus in phosphate-buffered saline (PBS) at an MOI of 1 plaque forming unit (PFU)/cell were placed on the cells for 30 min at room temperature. The infection D-MEM containing 5% of Tet-approved FBS, L-glutamax (10 mM), penicillin-streptomycin (10 mM) was then added. Tetracycline (10–30 μg/ml, Sigma-Aldrich) was placed in infection D-MEM media for induction of the A12L protein. Cell extracts were harvested at 24–48 hours post infection (hpi) by centrifugation (750 × g) for 5 min at 4°C, followed by three cycles of freezing and thawing to lyse the cells. Virus titers were conducted on BSC-40 cells, incubated at 37°C for 40 hours, and stained with 0.1% crystal violet solution in 30% ethanol.

### Transfection and marker rescue

In order to rescue the absence of A12L by plasmid-bourn A12L (pA12L), full-length of A12L ORF was placed right after an early/late synthetic promoter in pRB21 [21]. The same ORF were placed in TOPO TA cloning vector (Invitrogen) to drive A12L expression under its native promoter, which contains 233 upstream nucleotides (p233-A12L). To place A12L ORF in both pRB21 and TOPO vector, two different sets of primers were designed; pA12L-forward: 5'-CACTCCATGGATGGCGG ATAAAAAAAATTTAGCC and pA12L-reverse: 5'-CAGGATCCTTAATACATTCCCATATCCA GACAAC; p233-forward: 5'-ATGGCGGATAAAAAAAATTTAGCC and A12L-reverse: 5'-TTA ATACATTCCCATATCCAGACAAAATTCG. In order to construct A12L with abrogated cleavage at an N-terminal AG/A site (AG/A), the AG/A sites were altered into ID/I by site-directed mutagenesis kit (Stratagene) with a specific primer, which has the changed sequences at the residues 55–57 (underlined), 5'-CTTAATTCTCAAACAGATGTGACTATCGACATCTGTGATACAAAATCAAAGAGTTCA-3'. The AG/A site-mutated A12L was inserted in pRB21 vector.

For transfection of the plasmids into T-REx 293 cells, infection media of D-MEM medium was placed in new eppendorf tubes and mixed with 2 to 10 μg of DNA and 30 μl of the transfection reagent, DMRIE-C (Invitrogen). After vortexing the mixture, it was placed at room temperature for 20 min. and loaded on 6-well plates of ~ 60% confluent T-REx 293 cells. The cells were incubated at 37°C for 5–6 hours and infected by *vvtetO*A12L at an MOI of 1 PFU/cell for 24 hours. Virus titers were determined as described earlier.

### Electron microscopy

T-REx 293 cells were infected at an MOI of 1 PFU/cell with *vvtetO*A12L and harvested at 24 hpi by centrifugation (270 × g) at 4°C. The cell extracts were resuspended with 1X PBS, followed by incubation with fixative buffer (2% glutaraldehyde, 1.25% paraformaldehyde in 0.1 M cacodylate buffer [pH7.3]) for 2 hours at room temperature. Postfixation, ultrathin section, and staining were performed as described [22].

## Abbreviations

VV: Vaccinia virus; IV: Immature virus; IMV: Intracellular mature virus; *vvtetO*A12L:

A12L mutant virus; Tet: Tetracycline; TetO: Tetracycline operator.

## Competing interests

The author(s) declare that they have no competing interests.
